# Effect of dual glucose-dependent insulinotropic peptide/glucagon-like peptide-1 receptor agonist on weight loss in subjects with obesity

**DOI:** 10.3389/fendo.2023.1095753

**Published:** 2023-02-22

**Authors:** Isabella Zaffina, Maria Chiara Pelle, Giuseppe Armentaro, Federica Giofrè, Velia Cassano, Angela Sciacqua, Franco Arturi

**Affiliations:** ^1^ Unit of Internal Medicine, Department of Medical and Surgical Sciences, Magna Graecia University of Catanzaro, Catanzaro, Italy; ^2^ Geriatric Unit, Department of Medical and Surgical Sciences, Magna Graecia University of Catanzaro, Catanzaro, Italy; ^3^ Research Centre for the Prevention and Treatment of Metabolic Diseases (CR METDIS), Magna Graecia University of Catanzaro, Catanzaro, Italy

**Keywords:** GIP, GLP-1, obesity, weight loss, tirzepatide, GLP-1R agonists, GIP receptor agonists,

## Abstract

The occurrence of obesity is an increasing issue worldwide, especially in industrialized countries. Weight loss is important both to treat obesity and to prevent the development of complications. Currently, several drugs are used to treat obesity, but their efficacy is modest. Thus, new anti-obesity treatments are needed. Recently, there has been increased interest in the development of incretins that combine body-weight-lowering and glucose-lowering effects. Therefore, a new drug that simultaneously coactivates both the glucose-dependent insulinotropic polypeptide (GIP) receptor (GIPR) and the glucagon-like peptide-1 receptor (GLP-1R) has been developed. Tirzepatide, the first in this class, improves glycemic control by increasing insulin sensitivity and lipid metabolism as well as by reducing body weight. Combining the activation of the two receptors, greater improvement of β-cell function offers more effective treatment of diabetes and obesity with fewer adverse effects than selective GLP-1R agonists. In the present review, we discuss the progress in the use of GIPR and GLP-1R coagonists and review literature from *in vitro* studies, animal studies, and human trials, highlighting the synergistic mechanisms of tirzepatide.

## 1 Introduction

**Figure 1:**
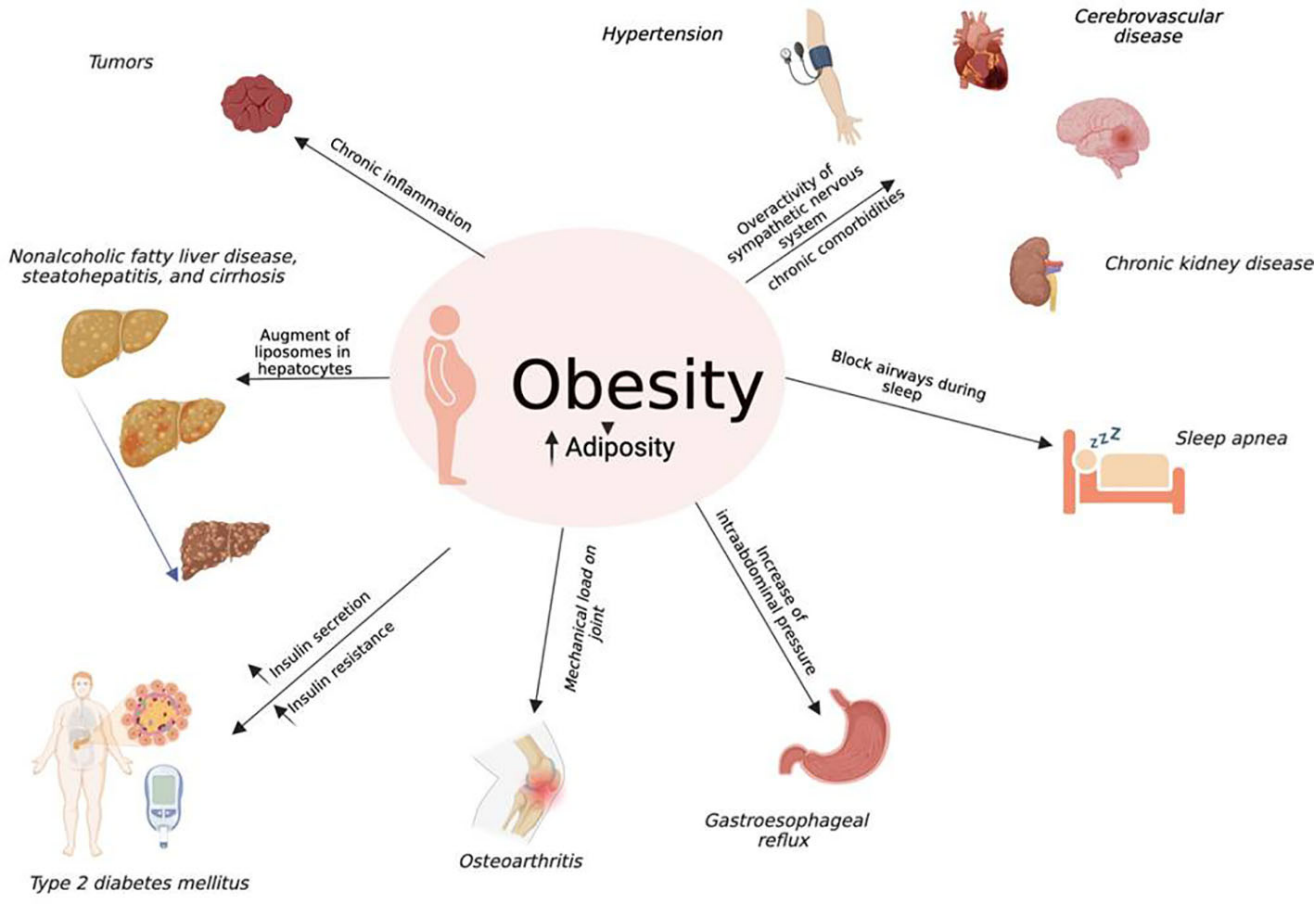
Obesity and co-morbidities: Obesity is distinguished by excess adiposity distributed to many body compartments. The increase of adiposity in pharyngeal soft tissue causes blocked airways during sleep, triggering obstructive sleep apnea; moreover, it causes both osteoarthritis, due to increased mechanical loading on the joints, and gastroesophageal reflux disease, due to an increase in intra-abdominal pressure. The obesity-related proinflammatory state with an increase in cytokines prompts insulin resistance, and together with insulin secretion that increases linearly with the BMI, supports dyslipidemia and T2DM. This obesity-induced chronic inflammation plays an endorsing role in cancer progression due to its promotion of a permissive microenvironment for neoplastic transformation. Furthermore, liposomes augment in hepatocytes that evolve in non-alcoholic fatty liver disease, steatohepatitis, and cirrhosis. Another consequence of obesity is chronic overactivity of the sympathetic nervous system that, together with the previously described consequences of chronic obesity, induces hypertension and increases the risk of heart disease, stroke, and chronic kidney diseases.

The World Health Organization (WHO) defines obesity as abnormal or excessive fat accumulation that presents a risk to health. Obesity is characterized by a body mass index (BMI) greater than 30 kg/m^2^, and it has rapidly become a global disease, with over four million deaths each year (1). The pathogenesis of obesity is multifaceted with environmental, socio-cultural, physiological, medical, behavioral, genetic, and epigenetic factors (2). Obesity is correlated with a wide variety of chronic diseases, including tumors, hypertension, type 2 diabetes mellitus (T2DM), cerebrovascular diseases, and chronic kidney disease (3). Obesity is characterized by excess adiposity that evolves gradually over time and is distributed to many body compartments. It is known that adipose tissue increases in pharyngeal soft tissue, causing blocked airways during sleep and triggering obstructive sleep apnea. Excess adiposity also determines both osteoarthritis, due to increased mechanical loading on the joints, and gastroesophageal reflux disease, due to an increase in intra-abdominal pressure. In obesity, an increase of macrophages and other immune cells in adipose tissue has been described, resulting in an increase in pro-inflammatory cytokines, promoting insulin resistance. Furthermore, insulin secretion increases linearly with the BMI, and insulin resistance favors dyslipidemia (4) and T2DM (2). This obesity-induced chronic inflammation plays an endorsing role in cancer progression due to its promotion of a permissive microenvironment for neoplastic transformation (5). Furthermore, liposomes augment in hepatocytes that evolve in non-alcoholic fatty liver disease, steatohepatitis, and cirrhosis. Another consequence of obesity is chronic overactivity of the sympathetic nervous system, which, together with the previously described consequences of chronic obesity, induces hypertension and increases the risk of heart disease, stroke, and chronic kidney dysfunction (2, 6, 7) (**Figure 1**). Thus, it is imperative to promote weight loss to reduce severe complications (8). The cornerstone of obesity management, which should be guided by a benefit-to-risk balance, comprises behavioral therapy (diet and lifestyle modifications), drugs, and bariatric surgery. Drug therapies, which should be considered for patients with a BMI of ≥30 kg/m^2^ and a BMI of ≥27 kg/m^2^ with weight-related comorbidities, stimulate satiety, reduce hunger, and/or reduce fat absorption or catabolism (9). Because the pathophysiology of obesity is complex, single-targeting agents have limited efficacy, suggesting that drug therapies that target multiple mechanisms are more effective than single-targeting agents (10). Among the medications approved for the long-term management of obesity, incretins represent appealing targets for inducing weight loss and preventing metabolic disorders. Glucagon-like peptide-1 (GLP-1) and glucose-dependent insulinotropic polypeptide (GIP) are two hormones responsible for the amplification of insulin secretion after nutrient consumption, and they have different actions (11). GLP-1 receptor agonist (GLP-1-RA) helps weight loss by suppressing appetite within the hypothalamus and inducing peripheral satiety by reducing gastric emptying, thus diminishing calorie intake (12). Similarly, GIP regulates energy balance through cell surface receptor signaling in adipose tissue and the brain (13). However, a recent study has shown that a dual GLP-1 and GIP receptor agonist achieves better glycemic control, insulin sensitivity, lipid metabolism, and body weight reduction (14) compared to either treatment alone. This mechanism may be related to synergistic action on β-cells (15), the hypothalamus, and the pro-opiomelanocortin (POMC) gene. In this review, we present the evidence supporting the use of a dual GIP/GLP-1 receptor agonist in the treatment of obesity, highlighting the physiological mechanisms and discussing the preclinical and clinical trials of this drug.

## 2 Physiological mechanism of the dual GIP/GLP-1 receptor agonist on weight loss: Evidence from preclinical studies

GLP-1 is a peptide that is synthesized by L cells of the intestine, and once GLP-1 is released, it binds to its receptor expressed in β-cells, α cells, the kidneys, the lungs, gastric mucosa, the heart, the brain, and immune cells (16). In addition to glucose control improvements, GLP-1-RAs, especially dulaglutide and semaglutide (17), stimulate significant weight loss by reducing gastric emptying, stimulating satiety, and decreasing food intake (18) by acting on peripheral and central receptors in the gut and brain (10). A recent study has demonstrated that liraglutide crosses the blood–brain barrier, reaching the arcuate nucleus (ARC), where GLP-1-RAs stimulate neurons that regulate appetite and express POMC and cocaine- and amphetamine-regulated transcripts (CART); liraglutide indirectly inhibits neurotransmission in neurons expressing neuropeptide Y (NPY) and agouti-related peptide (AgRP) *via* GABA-dependent signaling (19). GLP-1 RAs also reduce blood pressure, improve renal function, reduce chronic inflammation, reduce lipoprotein, reduce chylomicron, increase postprandial triglycerides, increase very-low-density lipoprotein cholesterol (VLDL-C), and increase free fatty acids (20). GIP, which is secreted by K cells of the duodenum and the proximal part of the small intestine, is the principal incretin hormone in humans, providing most of the incretin effect (21). GIP receptors (GIPRs) are distributed in the pancreas as well as in extra-pancreatic tissues, such as adipose tissue (22) (both white and brown adipose tissue (23)), the heart, the pituitary, the adrenal cortex (24), and some areas of the central nervous system (CNS) (25). GIP has long been considered a hormone that promotes obesity (26); GIP is excessively secreted after nutrient consumption, and it promotes fat deposition in adipose tissue (27). However, the role of GIP in weight control is not clear. In fact, on the one hand, it has been shown that GIP is involved in fat accumulation, and GIPR-deficient mice are resistant to obesity (28), but on the other hand, it has been demonstrated that chronic augmented GIP levels in a transgenic mouse model diminish diet-induced obesity as well as increase insulin sensitivity, glucose tolerance, and β-cell function (29). Moreover, scientific evidence has shown that GIP increases lipoprotein lipase (LPL) expression in adipocytes (30).

Some studies have demonstrated that the co-administration of GLP-1 and GIP acts synergistically on receptors that activate β-cells (15). In mouse models of diet-induced obesity, GLP-1-RA-induced weight loss has been demonstrated to be improved by the co-administration of a long-acting GIP analog (31). By using human islets isolated from patients with and without T2DM, Lupi et al. tested the effect of acute or extended incubation with GLP-1 and GIP, alone or in combination, and they reported that treatment with GLP-1 and GIP significantly enhanced glucose-stimulated insulin release but had no apparent synergistic effect in diabetic human islets (15); subsequently, prolonged incubation with the combination of GLP-1 and GIP improved insulin secretion in both diabetic and not diabetic human islets, and the combination of GLP-1 and GIP receptor agonists enhanced insulin gene expression and PDX-1 (β-cell differentiation factor) expression and increased β-cell survival. Furthermore, Delmeire et al. investigated the effects of GIP, with or without GLP-1, on the reactivity of healthy rat β-cells to glucose and GLP-1 (32), and they reported that a combination of GIP and GLP-1 at physiological concentrations increases reactivity to glucose, independent of insulin content. Therefore, the influence of GIP and GLP-1 during meals may be of particular relevance in shortening the postprandial phase of higher glucose levels and in stimulating β-cells for the next meal. Furthermore, Gault et al. demonstrated that a combined treatment of GIP and GLP-1 for 12 days in Swiss mice resulted in increased weight loss compared to treatment with exendin-4 alone but had no additional blood glucose regulation benefits (33). In addition, the co-administration of N-acetyl-GIP and GLP-1 analog for 14 days decreases plasma glucose levels and improves glucose tolerance in ob/ob mice, and there are no significant differences in plasma insulin, body weight, or food intake between the co-administration and either peptide alone (34). Previous studies have indicated that the co-administration of GIP and GLP-1 receptor agonists results in an additive effect on intracellular signaling pathways, as estimated by levels of intracellular cyclic adenosine monophosphate (cAMP) (35). The pharmacological effect of tirzepatide has been described in preclinical *in vitro* and *in vivo* studies. To date, tirzepatide, also known as LY3298176, is the only promising dual GIP/GLP-1 receptor agonist (23). To evaluate the effects of tirzepatide, many studies have been conducted in high-fat diet-fed, obese, insulin-resistant mice (23). Tirzepatide simultaneously stimulates GIP and GLP-1 receptors, which increases general insulin sensitivity, leading to improved glycemic control and better weight loss compared to GLP-1-RAs (36). Tirzepatide increases adiponectin and decreases serum alanine aminotransferase and lipoprotein biomarkers. Moreover, branched-chain amino acids (BCAAs) and their catabolic products, which are associated with the risk of obesity, insulin resistance, and T2DM, are significantly reduced by tirzepatide (37). Coskun et al. reported that tirzepatide stimulates cAMP accumulation with an efficacy similar to native GIP but weaker than GLP-1 in cell lines expressing recombinant GLP-1R and GIP-R *in vitro* (28). Moreover, Coskun et al. evaluated signaling in human pancreatic β-cells (ECN90) expressing both GIP and GLP-1 receptors, and they reported that these cells react to GIP or GLP-1 with a similar cAMP increase (28); in addition, tirzepatide stimulates higher levels of cAMP in ECN90 cells compared to GLP-1 or GIP alone. From the pharmacological point of view, signaling studies have shown that tirzepatide has mimetic effects of native GIP at the GIP receptor but shows bias at the GLP-1 receptor to promote cAMP generation over β-arrestin recruitment, which is in line with a weaker ability to induce GLP-1 receptor internalization compared to GLP-1 (38). Samms et al. demonstrated that tirzepatide has insulin-sensitizer effects in obese mice by improving insulin sensitivity (23). Interestingly, Samms et al. also showed that tirzepatide improves insulin action more effectively than GLP-1-RA and that this beneficial effect is independent of weight change through GIP-R agonism. The weight-independent insulin sensitization may suggest treatment durability in contrast to treatments that provide only weight-dependent effects. Recently, Samms et al. also demonstrated that tirzepatide affects the amino acid profile of metabolic organs in obese mice (39), an effect that occurs both in a weight-dependent and weight-independent manner. In a multicenter, randomized, double-blind, parallel-arm, phase 1 study, Heise et al. demonstrated that the administration of tirzepatide (15 mg) significantly improves the clamp disposition index from baseline to week 28 of treatment compared to semaglutide or a placebo; moreover, tirzepatide significantly reduces glucose excursions compared to a placebo, suggesting that the effect of tirzepatide is related to improvements in β-cell function, insulin sensitivity, and glucagon secretion (40). Furthermore, co-treatment with a GLP-1R agonist and a GIPR agonist results in more insulin sensitivity, glucose reduction, food intake reduction, and body weight reduction than either agonist alone in obese mice with type 2 diabetes (33). Tirzepatide, as highlighted in murine models, increases insulin sensitivity in a weight-dependent and weight-independent (through action on GIPR) manner, thereby stimulating metabolic pathways that oxidate glucose, lipids, and BCAAs. Thus, this mechanism, which avoids excessive nutrients, achieves metabolically active organs with a gain in insulin sensitivity and weight loss, providing long-lasting effects, unlike drugs, that act only in a weight-dependent manner (23). Recent data have indicated that treatment with tirzepatide stimulates the catabolism of BCAAs/branched-chain ketoacids (BCKAs) in brown adipose tissue (BAT). However, tirzepatide increases the level of amino acids in BAT to a level similar to that after cold exposure. Together, these results suggest that tirzepatide imitates the effect of cold exposure on the amino acid profile in BAT, resulting in improved insulin sensitization, increased metabolic rate, and increased weight loss (39).

**Figure 2:**
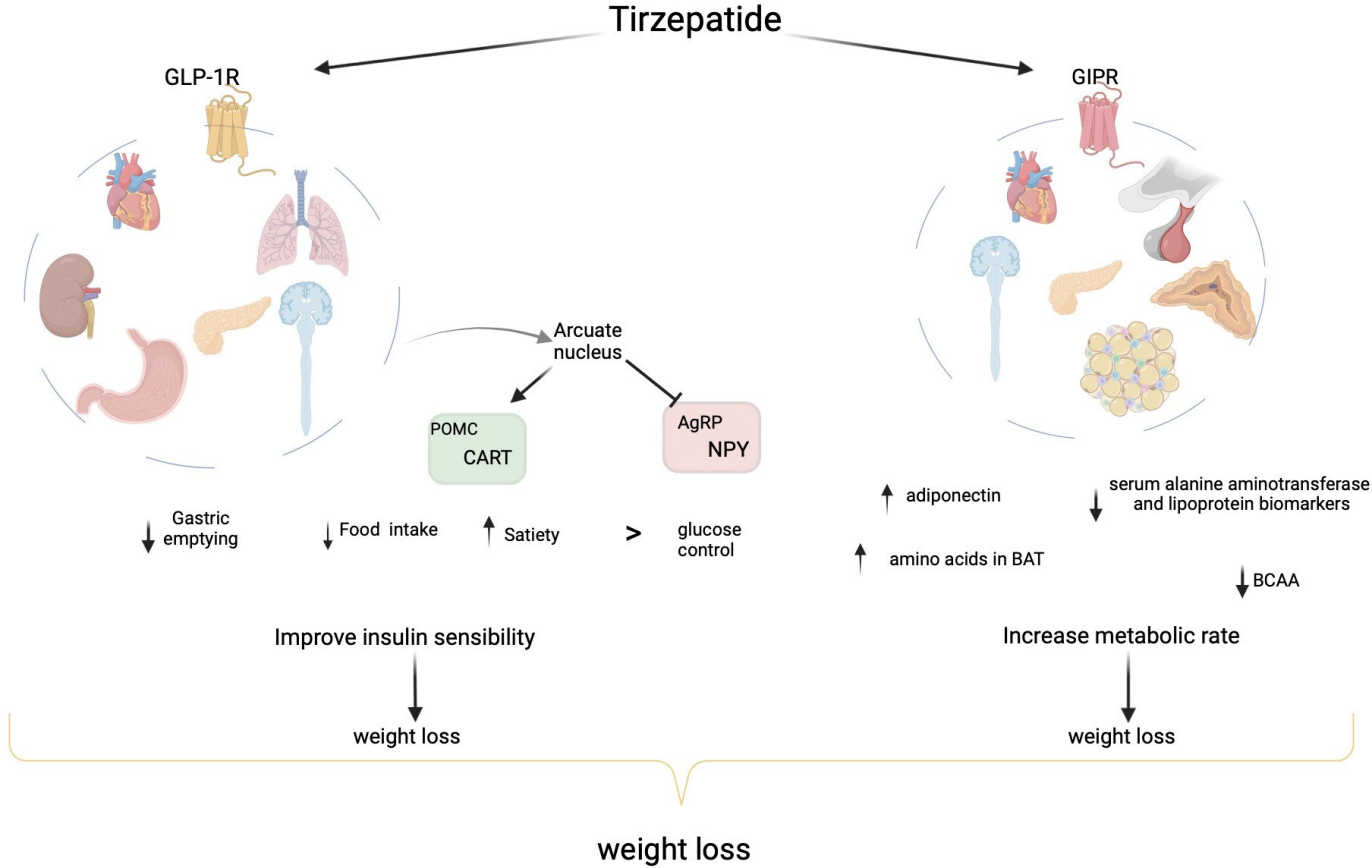
Physiological mechanism of incretins and a dual GIP/GLP-1 receptor agonist on weight loss. GLP-1, synthesized in the intestine, binds to its receptor, which is expressed in β-cells, α-cells, the kidneys, the lungs, gastric mucosa, the heart, and the brain. GLP-1-RA improves glucose control and stimulates significant weight loss by reducing gastric emptying, stimulating satiety, and decreasing food intake, and it acts on peripheral and central receptors in the gut and brain. GLP-1-RA reaches the arcuate nucleus, where it stimulates neurons that regulate appetite and express proopiomelanocortin (POMC) and cocaine- and amphetamine-regulated transcripts (CART), and it indirectly inhibits neurotransmission in neurons expressing neuropeptide Y (NPY) and agouti-related peptide (AgRP). GIP, secreted by K cells of the small intestine, binds to its receptor (GIPR) in the pancreas, adipose tissue (both white and brown adipose tissue), heart, pituitary, adrenal cortex, and some areas of the central nervous system (CNS). In a pharmacological context, GIP induces weight loss by stimulating satiety in the hypothalamus. Tirzepatide, which simultaneously stimulates GIP and GLP-1 receptors, increases general insulin sensitivity, improves glycemic control, and increases weight loss compared to GLP-1-RAs. Tirzepatide increases adiponectin and decreases serum alanine aminotransferase and lipoprotein biomarkers. Moreover, branched-chain amino acids (BCAAs) and their catabolic products are significantly reduced by tirzepatide. Tirzepatide imitates the effect of cold exposure on the amino acid profile in BAT, resulting in improved insulin sensitization, increased metabolic rate, and increased weight loss.

Though the exact mechanisms underlying GLP-1/GIP synergism are unclear, some hypotheses have suggested that GIP operates directly *via* the CNS by reducing food intake, increasing the anorexigenic action of GLP-1, or enhancing tolerability to GLP-1R agonists (13). The brain acts on both energy intake and expenditure, and control of energy balance is achieved by a complex mechanism involving the hypothalamus, hindbrain, amygdala, prefrontal cortex, and hippocampus. The ARC, a key site of the hypothalamus, receives direct signals from the periphery, and it contains both orexigenic neurons expressing Agouti-related peptide (AgRP)/NPY and anorexigenic neurons expressing POMC (27). In a pharmacological context, it has recently been shown that GIP induces weight loss by stimulating satiety in the hypothalamus, one of the sites of GIPR (27, 41), and chronic stimulus of GIPR in adipocytes desensitizes the receptor, causing antagonism (42). Finally, recent evidence has demonstrated that a supraphysiological dose of native GIP or the administration of a long-lasting GIP derivate decreases food intake and body weight (24, 27, 43, 44). A previous study has investigated the neuronal mechanism of the GLP-1/GIP receptor agonist on food intake and body weight through the intracerebroventricular (ICV) injection of these molecules to support data from studies conducted using the peripheral injection of GLP-1/GIP. ICV injection of the dual GLP-1/GIP receptor agonist reduces the doses of GLP-1 and GIP, activates hypothalamic neurons (c-fos), and increases anorectic POMC gene expression, while treatment with the same dose of GLP-1 and GIP alone does not achieve these results. Because these stimulated neurons represent a distinct population from POMC neurons, as indicated by a lack of co-localization, there may be a neuronal population aside from POMC cells in the arcuate nucleus (ARN) of the hypothalamus that is stimulated only by GLP-1 and GIP administration, the characteristics of which are still unknown (24). These neurons may be associated with anorexigenic POMC neurons and/or orexigenic AgRP neurons (24), or they may be associated with the brain, such as non-POMC anorexigenic neurons in the ARN, which have glutamatergic connections with the paraventricular nucleus of the hypothalamus (45). These neurons may play a role in behaviors such as drinking. Another study has demonstrated that GIP activation acts synergistically with GLP-1R activation, resulting in greater weight loss in mice than treatment with either agent alone, which correlates with both the enhanced suppression of calorie intake and an increase in energy expenditure (27). Recent literature suggests that central GLP-1 and GIP have a synergistic action on hypothalamic neurons with effects on food intake and body weight (24) (**Figure 2**).

## 3 Evidence from human studies

The first results relating to the synergistic effect of GIP and GLP-1RAs on humans came from small sample size studies in which the synergistic effect of an infusion of both molecules during an oral glucose tolerance test (OGTT) on insulin levels and glucose tolerance was observed. Subsequently, phase 1 and 2 studies were conducted, in which tirzepatide was compared to a placebo, and the outcomes were the efficacy and safety of tirzepatide and changes in baseline HbA1c after a few weeks. These studies showed that tirzepatide not only improved glycemic control but also reduced body weight. In a phase 1 double-blind randomized study, in which participants either received LY3298176 or a placebo, Coskun et al. demonstrated the considerable effect of weight loss in healthy subjects and patients with T2DM within only 4 weeks of treatment (28). Based on these results, phase 3 studies with tirzepatide were designed, and the SURPASS study was developed, which includes the following randomized controlled clinical trials: SURPASS-1, tirzepatide monotherapy; SURPASS-2, tirzepatide versus semaglutide; SURPASS-3, tirzepatide versus degludec; SURPASS-4, tirzepatide versus glargine in established CV disease; SURPASS-5, tirzepatide as a basal insulin add-on; SURPASS-6, tirzepatide versus insulin lispro in patients inadequately controlled on insulin glargine; and SURPASS J-mono, tirzepatide versus dulaglutide (11). Additional details on other ongoing trials with tirzepatide in patients with T2DM can be found at http://clinicaltrials.gov. In addition, a phase 3, multicenter, randomized, placebo-controlled study has been performed, in which adults with a BMI greater than 30 kg/m^2^ or 27 kg/m^2^ with at least one obesity-related complication, except T2DM, were recruited. In this study, tirzepatide was compared to a placebo (SURMOUNT-1). Several SURMOUNT studies in patients with obesity are ongoing (http://clinicaltrials.gov.).

In the SURPASS-1 study, which enrolled 705 non-target T2DM patients with diet and physical activity intervention, the efficacy, safety, and tolerability of tirzepatide and a placebo were compared. The cohort had a diabetes duration of 4.7 years, a mean glycated hemoglobin (HbA1c) of 7.9%, and a BMI of 31.9 kg/m^2^. At 40 weeks, a significant reduction in HbA1c proportional to the dosage of the drug used was observed (-1.87% with 5 mg of tirzepatide, -1.89% with 20 mg of tirzepatide, and -2.07% with 15 mg of tirzepatide versus +0.04% with the placebo). The weight loss ranged from 7 to 9.5 kg with tirzepatide, and there was a 10.1% greater decrease in weight with tirzepatide compared to the placebo. Severe hypoglycemia was not reported, and gastrointestinal events were the most frequent adverse effects in the tirzepatide group (46).

Tirzepatide was compared to once-weekly oral semaglutide in the SURPASS-2 study, an open-label, phase 3 clinical trial with a 40-week follow-up that enrolled T2DM patients with a BMI of ≥25 kg/m^2^ and an HbA1c of 7–10.5% despite taking at least a 1500 mg dose of metformin. In the SURPASS 2 study, the mean HbA1c levels were 8.28%, and the mean body weight was 93.7 kg. Tirzepatide, at all three doses, was non-inferior and superior to semaglutide at 1 mg in achieving the primary endpoint of HbA1c reduction. In addition, a greater effect of tirzepatide on dose-dependent body weight reduction versus semaglutide was observed (least-squares mean estimated treatment difference of -1.9 kg, -3.6 kg, and -5.5 kg for the three doses; p<0.001 for all comparisons) (47).

In the SURPASS-3 study, 1947 T2DM patients with poor glycometabolic control who were treated with metformin, with or without SGLT2 inhibitors, were enrolled, and the efficacy and safety of tirzepatide versus titrated insulin degludec were evaluated. At baseline, the mean HbA1c was 8.17 ± 0.91%, and the mean body weight was 94.3 ± 20.1 kg. The primary outcome was the reduction in HbA1c from baseline at 52 weeks. Tirzepatide, at the three doses, was superior to titrated insulin degludec, with greater reductions in HbA1c and body weight at week 52 as well as a lower risk of hypoglycemia; all three tirzepatide doses decreased body weight (-7.5 kg to -12.9 kg), whereas insulin degludec increased body weight by 2.3 kg (48).

In the SURPASS-4 study, the efficacy and safety of tirzepatide were evaluated in 2002 diabetic patients with established cardiovascular (CV) disease or a high risk of CV events who were treated with metformin, sulfonylurea, or SGLT2 inhibitors. The mean disease duration was 11.8 years, with a mean HbA1c of 8.52% and a mean body weight of 90.3 kg. The primary endpoint was a change in HbA1c from baseline to 52 weeks after treatment with 10 mg of tirzepatide, 15 mg of tirzepatide, or glargine. At 52 weeks, the mean changes in HbA1c were -2.43 ± 0.05%, -2.58 ± 0.05%, and -1.44 ± 0.03%, with 10 mg of tirzepatide and 15 mg of tirzepatide and glargine, respectively. Treatment with tirzepatide reduced body weight by 11.7 kg compared to glargine. Regarding the safety profile, fewer episodes of hypoglycemia (defined as glycemia < 54 mg/dl) occurred in the tirzepatide treatment group than in the glargine treatment group (6-9% vs. 19%), and this finding was more pronounced in patients not taking sulfonylureas (1-3% vs. 16%). However, more gastrointestinal adverse events, such as nausea, vomiting, diarrhea, and reduced appetite, occurred in the tirzepatide treatment group, requiring dose reduction (49).

The SURPASS-5 study was a phase 3, randomized, double-blind, placebo-controlled, 40-week, parallel, multicenter study comparing tirzepatide with a placebo in T2DM patients with inadequately controlled insulin glargine therapy with or without metformin. Tirzepatide was superior to the placebo in reducing the primary outcome, assessed as the difference in the percentage of reduction in HbA1c (10 mg: -1.53% difference, 97.5% CI-1.80 to -1.27%, p<0.001; 15mg: -1.47% difference; 97.5% CI-1.75to-1.20%, p<0.001). At week 40, a mean change in body weight from baseline proportional to the dosage used was observed as follows: -5.4 kg with 5 mg of tirzepatide, -7.5 kg with 10 mg of tirzepatide, -8.8 kg with 15 mg of tirzepatide, and 1.6 kg with the placebo (all p < 0.001). In addition, more gastrointestinal side effects occurred in the tirzepatide treatment group compared to the placebo group (50).

The SURPASS-6 study compared the efficacy and safety of tirzepatide to insulin lispro three times daily, with or without metformin, in a T2DM population, and the primary outcome was the change in HbA1c compared to baseline. No results are available because the study completion date was 1 November 2022

The SURPASS AP-Combo study was a randomized, phase 3, open-label trial comparing the effect of tirzepatide versus insulin glargine in T2DM patients on metformin with or without a sulfonylurea. The primary outcome was the change in HbA1c compared to baseline. No results are available because they are not yet published. The SURPASS J-mono study was a phase 3 trial of tirzepatide monotherapy compared to dulaglutide 0.75 mg in T2DM patients. The primary outcome was the change in HbA1c compared to baseline. Tirzepatide was superior to the comparator in reducing the primary outcome, assessed as the difference in the percentage of reduction in HbA1c (5 mg: -2.37% difference, 95% CI -1.27 to -0.90%, p<0.001; 10 mg: -2.55% difference; 95% CI -1.45 to -1.08%, p<0.001; 15 mg: -2.82% difference; 95% CI -1.71 to -1.35%, p<0.001). At week 52, a mean change in body weight from baseline was observed as follows: -5.8 kg with 5 mg of tirzepatide, -8.5 kg with 10 mg of tirzepatide, -10.7 kg with 15 mg of tirzepatide, and 0.5 kg with dulaglutide (all p < 0.001) (51).

The SURMOUNT-1 study was a phase 3, multicenter, randomized, placebo-controlled study, in which 2539 adults with a BMI greater than 30 kg/m^2^ or 27 kg/m^2^ and at least one obesity-related complication, except T2DM, were recruited, and tirzepatide was compared to a placebo. In this cohort, the mean body weight was 104.8 kg, and the mean BMI was 38.0 kg/m^2^. The coprimary endpoints were the percentage of reduction in BMI at 72 weeks from baseline and a reduction of 5% or more. At 72 weeks, a reduction in body weight proportional to the dosage of tirzepatide used was obtained as follows: a change of -15% (95% CI -15.9 to -14.2) was observed with 5-mg weekly doses of tirzepatide; a change of -19.5% (95% CI, -20.4 to -18.5) was observed with 10-mg weekly doses of tirzepatide; a change of -20.9% (95% CI, -21.8 to -19.9) was observed with 15-mg doses of tirzepatide; and a change of -3.1% (95% CI, -4.3 to -1.9) was observed with the placebo (p<0.001 for all comparisons to placebo). The percentage of patients who achieved a body weight reduction greater than or equal to 5% also correlated with the dose of tirzepatide as follows: 85% (95% CI, 82 to 89) with 5 mg of tirzepatide, 89% (95% CI, 86 to 92) with 10 mg of tirzepatide, 91% (95% CI, 88 to 94) with 15 mg of tirzepatide, and 35% (95% CI, 30 to 39) with the placebo. In addition, 50% (95% CI, 46 to 54) of patients in the 10-mg tirzepatide treatment arm and 57% (95% CI, 53 to 61) of those treated with 15 mg of tirzepatide had a body weight reduction of 20% or more compared to 3% (95% CI, 1 to 5) in the placebo group (p<0.001 for all comparisons to the placebo). Furthermore, the results also showed a change in body composition. A reduction in total body fat mass was demonstrated in the tirzepatide groups compared to the placebo group (33.9% versus 8.2%, respectively, for an estimated treatment difference relative to the placebo of -25.7 percentage points). Moreover, the weight reduction observed in patients treated with tirzepatide was accompanied by an improvement of all evaluated CV and metabolic risk factors, including waist circumference, fasting insulin level, systolic blood pressure, diastolic blood pressure, and lipid profile. In addition, most (>95%) of the patients in the tirzepatide groups who had prediabetes at baseline had converted to normoglycemia as compared to 62% of participants who received the placebo. The most common adverse effects reported were gastrointestinal symptoms that were mild to moderate in intensity and occurred during dose escalation (14) (**Table 1**).

**Table 1:** Randomized controlled trials of tirzepatide in obese patients.

Study	Population	Sample size	Intervention	Outcome	Results
**SURPASS-1** **NCT03954834**	Patients over 18 years old with T2DM, HbA1c between ≥7.0% and ≤9.5%, and BMI ≥23kg/m^2^	478 total Tirzepatide 5 mg (n=121), Tirzepatide 10 mg (n=121), Tirzepatide 15 mg (n=121) vs Placebo (n=115)	Tirzepatide 5 mg vs. Tirzepatide 10 mg vs. Tirzepatide 15 mg vs. Placebo	Primary outcome: Change in HbA1c from baseline Secondary outcome: Change in body weight from baseline*	In the Tirzepatide5 mg group, body weight decreased by 7 kg. In the Tirzepatide10 mg group, body weight decreased by 7.8 kg. In the Tirzepatide15 mg group, body weight decreased by 9.5 kg. In the Placebo group, body weight decreased by 0.7 kg.
**SURPASS-2** **NCT03987919**	Patients over 18 years old with T2DM, HbA1c between ≥7.0% and ≤10.5%, and BMI ≥25kg/m^2^	1879 total Tirzepatide 5 mg (n=471), Tirzepatide 10 mg (n=469), Tirzepatide 15 mg (n=470), and Semaglutide 1 mg (n=469)	Tirzepatide 5 mg vs. Tirzepatide 10 mg vs. Tirzepatide 15 mg vs. Semaglutide 1 mg	Primary outcome: Change in HbA1c from baseline Secondary outcome: Change in body weight from baseline*	In the Tirzepatide 5 mg group, body weight decreased by 7.8 kg. In the Tirzepatide 10 mg group, body weight decreased by 10.3 kg. In the Tirzepatide 15 mg group, body weight decreased by 12.4 kg. In the Semaglutide 1 mg group, body weight decreased by 6.2 kg.
**SURPASS-3** **NCT03882970**	Patients over 18 years old with T2DM, HbA1c between ≥7.0% and ≤10.5%, and BMI ≥25kg/m^2^	1444 total Tirzepatide 5 mg (n=359), Tirzepatide 10 mg (n=361), Tirzepatide 15 mg, and (n=359) Insulin Degludec (n=365)	Tirzepatide 5 mg vs. Tirzepatide 10 mg vs. Tirzepatide 15 mg vs. Insulin Degludec	Primary outcome: Change in HbA1c from baseline Secondary outcome: Change in body weight from baseline*	In the Tirzepatide 5 mg group, body weight decreased by 7.5 kg. In the Tirzepatide 10 mg group, body weight decreased by 10.7 kg. In the Tirzepatide 15 mg group, body weight decreased by 12.9 kg. In the Insulin Degludec group, body weight increased by 2.3 kg.
**SURPASS-4** **NCT03730662**	Patients over18 years old with T2DM, HbA1c between ≥7.0% and ≤10.5%, and BMI ≥25kg/m^2^	2002 total Tirzepatide 5 mg (n=329), Tirzepatide 10 mg (n=330), Tirzepatide 15 mg (n=338), and Insulin Glargine (n=1005)	Tirzepatide 5 mg vs. Tirzepatide 10 mg vs. Tirzepatide 15 mg vs. Insulin Glargine	Primary outcome: Change in HbA1c from baseline Secondary outcome: Change in body weight from baseline *	In the Tirzepatide 5 mg group, body weight decreased by 7.1 kg. In the Tirzepatide 10 mg group, body weight decreased by 9.5 kg. In the Tirzepatide 15 mg group, body weight decreased by 11.7 kg. In the Insulin Glargine group, body weight increased by 1.9 kg.
**SURPASS-5** **NCT04039503**	Patients over 18 years old with T2DM treated with insulin glargine (U100) once daily, with or without metformin ≥3 months prior to screening visit, HbA1c between ≥7.0% and ≤10.5%, and BMI ≥23kg/m^2^	475total Tirzepatide 5 mg (n=116), Tirzepatide 10 mg (n=119), Tirzepatide 15 mg (n=120), and Placebo (n=120)	Tirzepatide 5 mg vs. Tirzepatide 10 mg vs. Tirzepatide 15 mg vs. Placebo	Primary outcome: Change in HbA1c from baseline Secondary outcome: Change in body weight from baseline*	In the Tirzepatide 5 mg group, body weight decreased by 6.2 kg. In the Tirzepatide 10 mg group, body weight decreased by 8.2 kg. In the Tirzepatide 15 mg group, body weight decreased by 10.9 kg. In the Placebo group, body weight increased by 1.7 kg.
**SURPASS-6** **NCT04537923**	Patients over 18 years old with T2DM, HbA1c between ≥7.5% and ≤11%, treated for at least 90 days prior to the day of screening with once or twice daily basal insulin with or without a stable dose of metformin ≥1500 mg/day and up to maximum approved dose per country-specific approved label, sulfonylureas or dipeptidyl peptidase 4 inhibitors, and BMI ≥23kg/m^2^ ≤45 kg/m² and stable weight (± 5%) for at least 90 days	1428 total	Tirzepatide 5 mg with Insulin Glargine (U100) vs. Tirzepatide 10 mg with Insulin Glargine (U100) vs. Tirzepatide 15 mg with Insulin Glargine (U100) vs. Insulin Lispro (U100) with Insulin Glargine (U100)	Primary outcome: Change in HbA1c from baseline Secondary outcome: Change in body weight from baseline*	No results available because study completion date was November 1, 2022.
**SURPASS J-mono** **NCT03861052**	Patients over 20 years old with T2DM, HbA1c between ≥7.0% and ≤10.5% for those who are oral antihyperglycemic medication (OAM)-naïve at screening, between ≥6.5% and ≤9.0% at screening, and between ≥7.0% and ≤10.5% at baseline for patients who have been taking OAM monotherapy at screening, and BMI ≥23kg/m^2^	636total Tirzepatide 5 mg (n=159), Tirzepatide 10 mg (n=158), Tirzepatide 15 mg (n=160), and Dulaglutide 0.75 mg (n=159)	Tirzepatide 5 mg vs. Tirzepatide 10 mg vs. Tirzepatide 15 mg vs. Dulaglutide 0.75 mg	Primary outcome: Change in HbA1c from baseline Secondary outcome: Change in body weight from baseline*	In the Tirzepatide 5 mg group, body weight decreased by 5.8 kg. In the Tirzepatide 10 mg group, body weight decreased by 8.5 kg. In the Tirzepatide 15 mg group, body weight decreased by 10.7 kg. In the Dulaglutide 0.75 mg group, body weight decreased by 0.5 kg.
**SURPASS AP-Combo** **NCT04093752**	Patients over 18 years old with T2DM, HbA1c between ≥7.5% and ≤11% treated with stable metformin, with or without a sulfonylurea, for at least 2 months, stable weight ≥ 3 months, BMI ≥ 23 kilograms per meter squared	917 total	Tirzepatide 5 mg vs. tirzepatide 10 mg vs. tirzepatide 15 mg vs. Insulin Glargine	Primary outcome: Change in HbA1c from baseline Secondary outcome: Change in body weight from baseline*	No results available because not yet published.
**SURMOUNT-1** **NCT04184622**	Patients over 18 years old with BMI ≥30 kg/m² or ≥27 kg/m² and previous diagnosis with at least one of the following comorbidities: hypertension, dyslipidemia, obstructive sleep apnea, or cardiovascular disease. History of at least one unsuccessful dietary effort to lose body weight	2539 total Tirzepatide 5 mg, Tirzepatide 10 mg, Tirzepatide 15 mg, and Placebo	Tirzepatide 5 mg vs. Tirzepatide 10 mg vs. Tirzepatide15 mg vs. Placebo	Primary outcomes: 1. Percent change in body weight from baseline 2. Percentage of participants who achieve ≥5% body weight reduction*	In the Tirzepatide 5 mg group, body weight decreased by 15%. In the Tirzepatide 10 mg group, body weight decreased by 19.5%.In the Tirzepatide 15 mg group, body weight decreased by 20.9%.In the Placebo group, body weight decreased by 3.1%. The percentage of participants who had a weight reduction of 5% or more was 85% in the Tirzepatide 5 mg group, 89% in the Tirzepatide 10 mg group, 91% in the Tirzepatide 15 mg group, and 35% in the Placebo group.

* Additional details on other secondary outcomes other than the change in body weight can be found at http://clinicaltrials.gov as NCT03954834 (SURPASS-1), NCT03987919 (SURPASS-2), NCT03882970 (SURPASS-3), NCT03730662 (SURPASS-4), NCT04039503 (SURPASS-5), NCT04537923 (SURPASS-6), NCT04255433 (SURPASS-CVOT), NCT03861052(SURPASS J-mono), NCT04093752 (SURPASS-AP-Combo), and NCT04184622 (SURMOUNT-1).

However, the benefits of tirzepatide are not limited only to weight loss and glycaemic control. Interesting data arose from sub- and meta-analyses of RCTs regarding renal protection, cardiovascular safety, non-alcoholic fatty liver disease (NAFLD), and cognitive impairment. A *post hoc* analysis of data from SURPASS-4 showed that tirzepatide is effective in reducing the risk of worsening renal function compared with insulin glargine. Treatment with tirzepatide slowed the decline in the estimated glomerular filtration rate (eGFR) and reduced the urine albumin-creatinine ratio (UACR) in a clinically meaningful way compared with insulin glargine. Participants receiving tirzepatide showed a significantly lower occurrence of the composite kidney endpoint defined as a decline in eGFR of ≥40%, the new onset of macroalbuminuria defined by a UACR of >300 mg/g, and the occurrence of end-stage renal weeks (52). There are also encouraging data on the CV safety of this drug. A meta-analysis including the seven SURPASS trials compared tirzepatide with control groups on the occurrence of 4-point MACE (MACE 4P, cardiovascular death, myocardial infarction, stroke, and hospitalized unstable angina) and confirmed the CV safety of this drug. In fact, tirzepatide was not associated with an increased risk of MACE 4P and all-cause mortality compared with controls (53). Treatment with once-weekly tirzepatide at the doses of 5 mg, 10 mg, and 15 mg, with controlled treatment exposure of up to 104 weeks, was not associated with an increased risk for cardiovascular events in people with T2DM (53). However, a large prospective cardiovascular outcome trial (SURPASS-CVOT, Clinical Trials.gov NCT04255433) is ongoing. In this trial, tirzepatide is compared with dulaglutide, a pure GLP-1 receptor agonist that demonstrated a significant reduction of cardiovascular risk in the REWIND study (54). The objective is to demonstrate efficacy in the reduction of cardiovascular risk and evaluate the cardiovascular safety of tirzepatide. In addition, tirzepatide is also effective in the treatment of emerging conditions such as NAFLD. A sub-study of the randomized phase 3 SURPASS-3 trial evaluated the changes in liver fat content (LFC), abdominal subcutaneous adipose tissue (ASAT), and volume of visceral adipose tissue (VAT) in patients treated with tirzepatide compared to patients treated with insulin degludec. Tirzepatide showed a significant reduction in LFC and VAT and in ASAT volumes compared with insulin degludec in this subpopulation of patients with type 2 diabetes in the SURPASS-3 study (55). Lastly, tirzepatide has been shown to play a neuroprotective role in cellular and animal models. Indeed, in animal models with mild traumatic brain injury, the administration of tirzepatide has been shown to fully restore visual and spatiotemporal memory deficits. These results suggest a neuroprotective role played by tirzepatide in cognitive impairment. However, further studies on humans are needed to better understand and define the mechanisms of action of this drug in the central nervous system (56).

## 4 Adverse effects: Tirzepatide vs GLP-1 RAs

In clinical trials, the main cause of discontinuation of tirzepatide was adverse events that seem to be qualitatively and quantitatively comparable to those reported in patients treated with GLP-1 Ras, and they are more common at the higher dose of tirzepatide, supporting a dose-dependent safety profile (47, 48). The main adverse effects described in clinical trials employing tirzepatide were gastrointestinal, such as nausea, diarrhea, decreased appetite, delayed gastric emptying, vomiting, acid reflux, and constipation (57). Moreover, sinus tachycardia (58), transient increases in the mean pulse rate, acute kidney injury due to dehydration, and hypersensitivity reactions at the injection site have been described. Additional events were acute pancreatitis (59), although cases were not serious and no differences between tirzepatide and GLP1 RAs were demonstrated (60), and biliary tract cholelithiasis and cholecystitis (61). The eye may also be affected, especially in patients with preexisting diabetic retinopathy, in whom those symptoms may briefly deteriorate if their glycemic control rapidly improves (62). Instead, hypoglycemia occurs in a small percentage of patients, but compared with GLP-1 RA, tirzepatide has a dose-dependent risk of hypoglycemia (60, 63).

## 5 Future perspectives

The therapeutic *armamentarium* for the management of patients with obesity is undergoing an important revolution. In recent years, other dual or triple receptor agonists have been developed by the pharmaceutical industry besides tirzepatide (64). Among them, triple GLP-1/GIP/GCC receptor agonists are of particular interest (65, 66) and studies are currently underway to investigate the efficacy and safety of these combinations. LY3437943 is a novel triple agonist peptide at the glucagon receptor (GCGR), GIPR, and GLP-1R (65). The efficacy of the tri-agonists derives from the anorectic and insulinotropic activities of GLP-1 and GIP and the energy expenditure induction of glucagon (67). Preclinical studies conducted in C57/Bl6 diet-induced obese (DIO) mice demonstrated that LY3437943 is effective in reducing body weight and calorie intake. Moreover, similarly to the dual GIPR/GLP-1R agonist, LY3437943 reduced blood glucose and plasma insulin, suggesting potential improvements in insulin sensitivity. Then, in obese mice, the administration of LY3437943 decreased body weight and improved glycemic control. A single ascending dose, first-in-human phase 1 study to assess the safety and pharmacokinetic profile of LY3437943 was performed on 47 healthy participants (ClinicalTrials.gov, NCT03841630). Treatment with LY3437943 resulted in a decrease in mean body weight from baseline at all dose levels except the lowest dose of 0.1 mg (65). In another phase 1b human study conducted in the USA (ClinicalTrials.gov, NCT04143802), LY3437943 was compared with a placebo and 1.5 mg dulaglutide in patients with T2DM, demonstrating a dose-dependent body weight reduction in the LY3437943 group (68). Therefore, the administration of LY3437943 induces a decrease in body weight and shows a safety and tolerability profile similar to other incretins in humans as well. Similar results have been obtained using SAR441255, another GLP-1R/GIPR/GCGR tri-agonist once-daily subcutaneous injection. This triple agonist has demonstrated superiority to dual coagonists (GLP-1R/GIPR) and mono-agonists (GLP-1) in reducing body weight (reduction of 25% in 4 weeks), improving glycemic control, and reducing hepatic steatosis in rodent models. SAR441255 also reduced body weight (~12%) in DIO monkeys after 7 weeks, confirming its efficacy in mammalian species (66, 69). However, although the data obtained from preclinical studies involving a relatively small number of healthy subjects are promising, further comparative trials in larger populations of people with T2DM and obesity are required to confirm the therapeutic potential of this new GLP-1, GIP, and GCG receptor tri-agonist, particularly in comparison with existing selective GLP-1 and dual GLP-1/GIP receptor agonists.

## 6 Conclusion

Obesity and T2DM are strongly associated, sharing pathophysiological mechanisms and long-term complications. Because achieving weight loss with lifestyle change alone in individuals with obesity is difficult, various approaches, from surgery to drug therapy, have been proposed over the years. However, weight loss is not always satisfactory and is often not maintained over time, indicating that continual research is needed. The alarming worldwide increase in obesity requires the development of new molecules that increase patient compliance by reducing the frequency of administration, facilitating intake, and reducing the adverse effects of treatments. Dual GIP/GLP-1 receptor agonists, such as tirzepatide, are promising molecules that not only improve glycemic control but also reduce body weight. In the spring of 2022, the Food and Drug Administration (FDA) approved tirzepatide for the management and treatment of T2DM, and it has also recently been approved in Europe. Given the results of the SURMOUNT-1 trial on weight loss, the introduction of tirzepatide in possible drug therapies against obesity not associated with diabetes is desirable.

## Author contributions

IZ, MP, GA, FG, VC, AS, and FA participated in the study conceptualization. IZ and MP participated in the methodology. IZ, MP, GA, FG, VC, AS, and FA participated in the original draft preparation. IZ, MP, and FA participated in writing, reviewing, and editing. AS and FA participated in study supervision. All authors read and agreed to the published version of the manuscript.
